# Challenge to the concept of surgical dose-response for moderate to large angle exotropia

**DOI:** 10.1007/s10384-026-01344-1

**Published:** 2026-04-25

**Authors:** Soh Youn Suh, Qingyu Meng, Ami Tamhaney, Joseph L. Demer

**Affiliations:** 1 Department of Ophthalmology, University of California, Los Angeles, CA USA; 2 Stein Eye Institute, UCLA, 100 Stein Plaza, Los Angeles, CA 90095-7002 USA; 3 David Geffen Medical School, University of California, Los Angeles, CA USA; 4 Department of Neurology, University of California, Los Angeles, CA USA; 5 Neuroscience Interdepartmental Program, University of California, Los Angeles, CA USA; 6 Bioengineering Department, University of California, Los Angeles, CA USA; 7 Peking University People’s Hospital, Beijing, China

**Keywords:** Exotropia, Surgical dose-response, Three-muscle strabismus surgery

## Abstract

**Purpose:**

To determine whether the effect of three muscle surgery for moderate to large angle exotropia can be better predicted by surgical dosage, or by pre-operative exotropia angle. We examined cases where identical surgical dose was performed for a wide range of preoperative angles 30 to 55Δ.

**Study design:**

Retrospective, interventional case study.

**Methods:**

Patients with exotropia 30–55Δ who underwent bilateral lateral rectus (BLR) muscle recession 7 mm and 4 mm unilateral medial rectus (UMR) muscle plication between 2014 and 2024 were included. Surgical effect was analyzed by linear regression against the amount of preoperative exotropia, as well as total millimeters of recession and plication. Success was defined as less than 10Δ exotropia or 5Δ esotropia.

**Results:**

Thirty-four patients were included with 32 ± 27 (standard deviation, SD) years mean age. Mean preoperative exotropia at distance was 42 ± 6Δ (range 30–55Δ), and 40 ± 16Δ (range 6–70Δ) at near. Surgical effect varied from 29Δ to 75Δ, averaging 42 ± 10Δ, giving a mean dose response of 2 ± 1Δ/mm. Linear regression showed that average surgical effect was equal to pre-operative deviation, accounting for 34–36% of variation in surgical effect. Success rate of this operation for all exotropia angles was 76% at 2 months mean followup.

**Conclusions:**

Surgical effect for moderate to large angle exotropia does not strongly depend on surgical dose, so that successful outcomes measured 2 months post-operatively are obtained by performing a numerically identical procedure regardless of the pre-operative angle exceeding 30Δ. Since outcome is attributable more to the biological response than to amount of surgery, pre-operative measurements require only sufficient precision to confirm that the angle is moderate to large.

## Introduction

Based on the belief that the effect of strabismus surgery depends on the its quantitative dosing, the amount of surgical muscle manipulation has been traditionally adjusted to the preoperative strabismus angle [1]. Recommended dosing tables for strabismus surgery have been propounded on the assumption that prism diopter correction per millimeter of recession or resection is predictable. An early dose-response curve for bilateral medial recession for infantile esotropia was published in 1985 [2]. However, the dose-response graph demonstrated significant variability of surgical effects, especially for esotropia exceeding 30Δ.

Exotropia is notorious for its high recurrence rate; multiple studies have been performed to investigate factors possibly affecting surgical outcomes [3–13]. Currently there is little consensus on either unilateral or bilateral surgery, nor on the number of horizontal extraocular muscles, and the amount of surgery to be performed on them, for treating large angle exotropia. Some surgeons advocate performing maximal or supramaximal doses on two horizontal muscles [14–17], while others recommend three-muscle surgery to avoid lateral incomitance [17–22].

Some studies suggest that the results of strabismus surgery may not be closely related to the amount of surgery performed. Tran et al. suggest that e preoperative deviation was the most powerful predictor of the surgical outcome [23]. By reanalyzing the data of Tran et al., Archer concludes that the preoperative deviation was better correlated with surgical outcome than the actual amount of surgery [24]. However, such analyses are confounded by intentional modification of the surgical dose based on pre-operative strabismus magnitude. The ideal test of an hypothesized relationship between surgical dose and response would require fixed surgical dosing independent of pre-operative strabismus angle. The published literature does not contain such data for exotropia.

The current study was conducted subject to the forgoing ideal condition, examining a surgical series where identical surgical dosing was administered for a range of pre-operative exotropia angles. We asked whether the effect of three horizontal muscle surgery for moderate to large angle exotropia can be better predicted by surgical dose versus pre-operative angle.

## Methods

This study was approved by the International Review Board of the University of California, Los Angeles, and followed the tenets of Declaration of Helsinki (IRB #10-001528). Records were reviewed of all consecutive patients with exotropia who underwent bilateral 7mm lateral rectus muscle recession and unilateral 4mm medial rectus muscle plication by two surgeons (J.L.D. and S.Y.S) at the Stein Eye Institute, University of California Los Angeles (UCLA), between 2014 and 2024. Patients were required to have at least 2 postoperative follow up visits to evaluate the shift in ocular alignment between the 1st and 2nd postoperative visits. A second postoperative visit was chosen because many patients live far from UCLA and would not return for further follow ups beyond the 2nd postoperative visit. The 1st and 2nd postoperative visits were usually performed at 1 week and 6–8 weeks after surgery, respectively. However, there was greater variability in the 2nd postoperative visits due to some patients being unable to meet their scheduled visit and and instead following up opportunistically.

Patients were excluded if the strabismus was due to paresis/paralysis or restriction, or when they had any prior muscle surgery, orbital trauma, vertical transposition of rectus insertions, history of prior strabismus surgery, or neurologic disorders, such as cerebral palsy or seizure disorders.

Patients underwent complete ophthalmological examination prior to the surgery. Visual acuity (VA) was measured using Snellen or Allen picture optotypes. Binocular alignment was measured pre- and postoperatively using the alternate prism cover testing at distance (4 m) and near (33 cm). The Krimsky test was used in patients who were too young or showed limited cooperation for the alternate prism cover test. Simultaneous prism cover test was not performed. Patients were considered to have lateral incomitance if the exotropia varied by at least 5Δ between central and lateral gazes.

It has long been the practice of the operating surgeons to employ the following surgical strategy for all cases of moderate to large angle exotropia. Surgery was performed using standard technique under general anesthesia using the same surgical dose regardless of the angle of preoperative exotropia. The non-dominant eye was usually plicated. No postoperative adjustments were made. All patients were examined within 21 days of surgery for the first postoperative visit and at variable times later for the second postoperative visit. Successful surgical outcome was defined when postoperative deviation was less than 10Δ exotropia or 5Δ esotropia. Linear regression was used to evaluate the relationship between preoperative exotropia and surgical effect using GraphPad Prism version 10 (GraphPad Software). Goodness of fit of the regressions was assessed using the coefficient of determination (*R*^2^), which indicates the fraction of variance of the best fitting linear response attributable to the input variable.

## Results

A total of 54 cases of bilateral 7 mm lateral rectus muscle recession combined with unilateral 4mm medial rectus muscle plication were reviewed. Of these, 19 were excluded: 6 had undergone simultaneous vertical muscle surgery or vertical transposition of rectus insertions, 6 had neurological disorders, 7 had only one postoperative visit, and 1 had undergone previous strabismus surgery. Included were 34 patients (23 men and 11 women) of mean age 32 ± 27 years (standard deviation, SD, range, 1–78 years) and median age 18 years. Nine patients had poorly controlled intermittent exotropia and 25 patients had constant exotropia. Mean preoperative exotropia was 42 ± 6Δ (range 30–55Δ) at distance and 40 ± 16Δ (range 6–70Δ) at near.

Preoperatively, no patient demonstrated limitation to adduction. Preoperative lateral incomitance was observed in 6 among the 33 patients (18%) whose measurements were available in lateral gazes. Among 33 patients whose VA was measurable, two had 20/40 unilateral amblyopia, while all others had bilaterally symmetric best corrected visual acuity of at least 20/25. Fix and follow movements were bilaterally symmetric in a 1 year-old patient, whose exotropia started around 11 months of age as intermittent exotropia and surgery was performed when the patient was 17 months old.

**Fig. 1:**
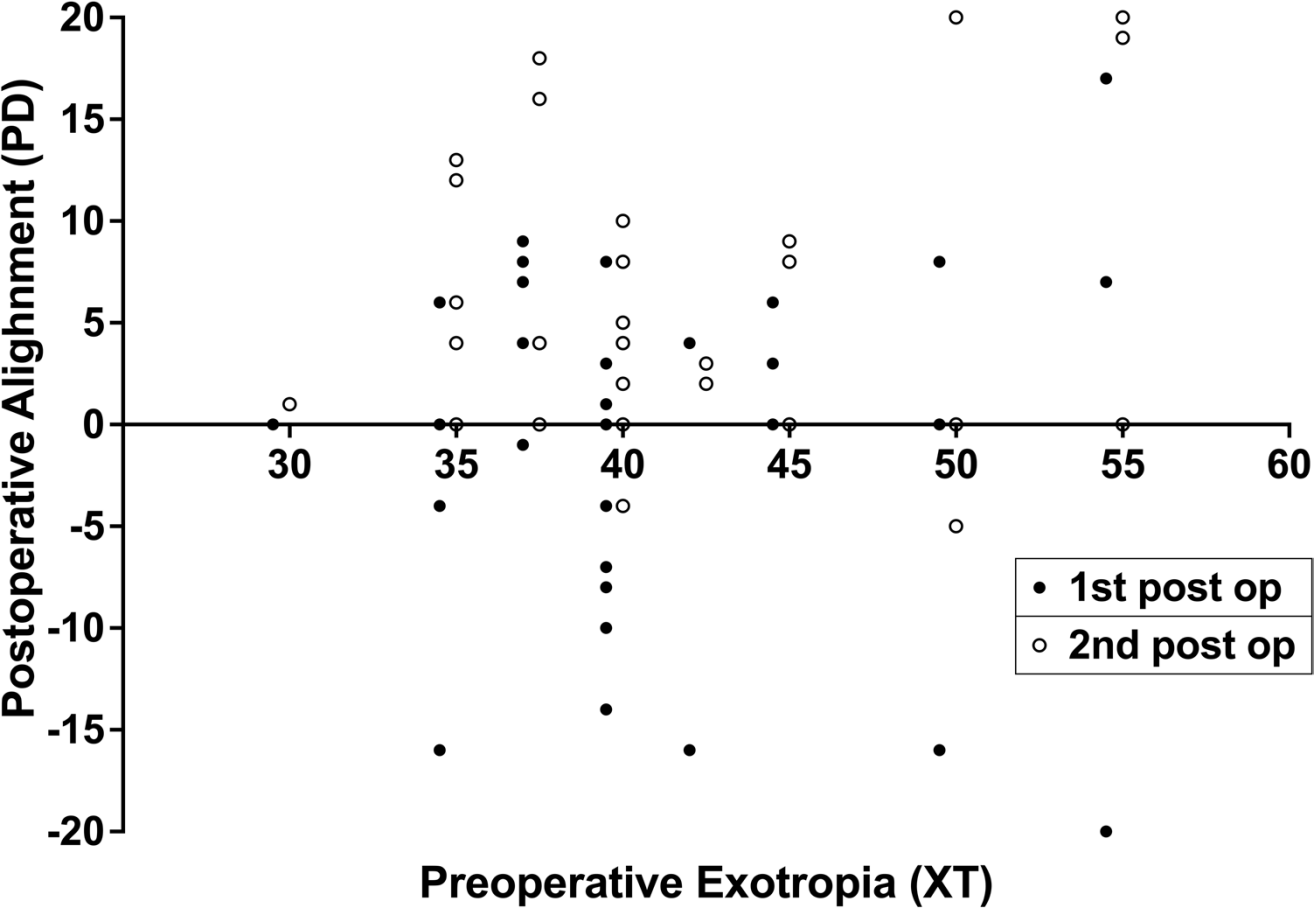
Pre- and postoperative exotropia at distance. Mean postoperative exodeviation was -0.5 ± 8.6Δ and 5.8 ± 6.7Δ at first and second postoperative visit, respectively. Symbols of the first postoperative visit are nudged to the left to avoid overlap

The first post-operative visit occurred at an average of 7 ± 4 (range 1–21) days after surgery, and the second at 60 ± 31 (range 22–183) days. At the first postoperative visit, exodeviation averaged – 1 ± 9 Δ (range − 20 to 17Δ) at distance and 2 ± 7 Δ (range − 18 to 16D) at near (Fig. 1). At the second postoperative visit, exodeviation averaged 6 ± 7Δ (range − 5 to 20Δ) at distance and 8 ± 9Δ (range − 4 to 25Δ) at near.

There was lateral incomitance at the first postoperative visit in 5 (19%) of the 26 patients where such measurements were available. At the second postoperative visit, lateral incomitance was observed in 5 (15%) of 34 patients. Success rates were 74% and 75% at first and second postoperative visits, respectively. Consecutive esotropia exceeding 5Δ was present in 8 patients (24%) at first postoperative visit but resolved in all by second visit.

**Fig. 2:**
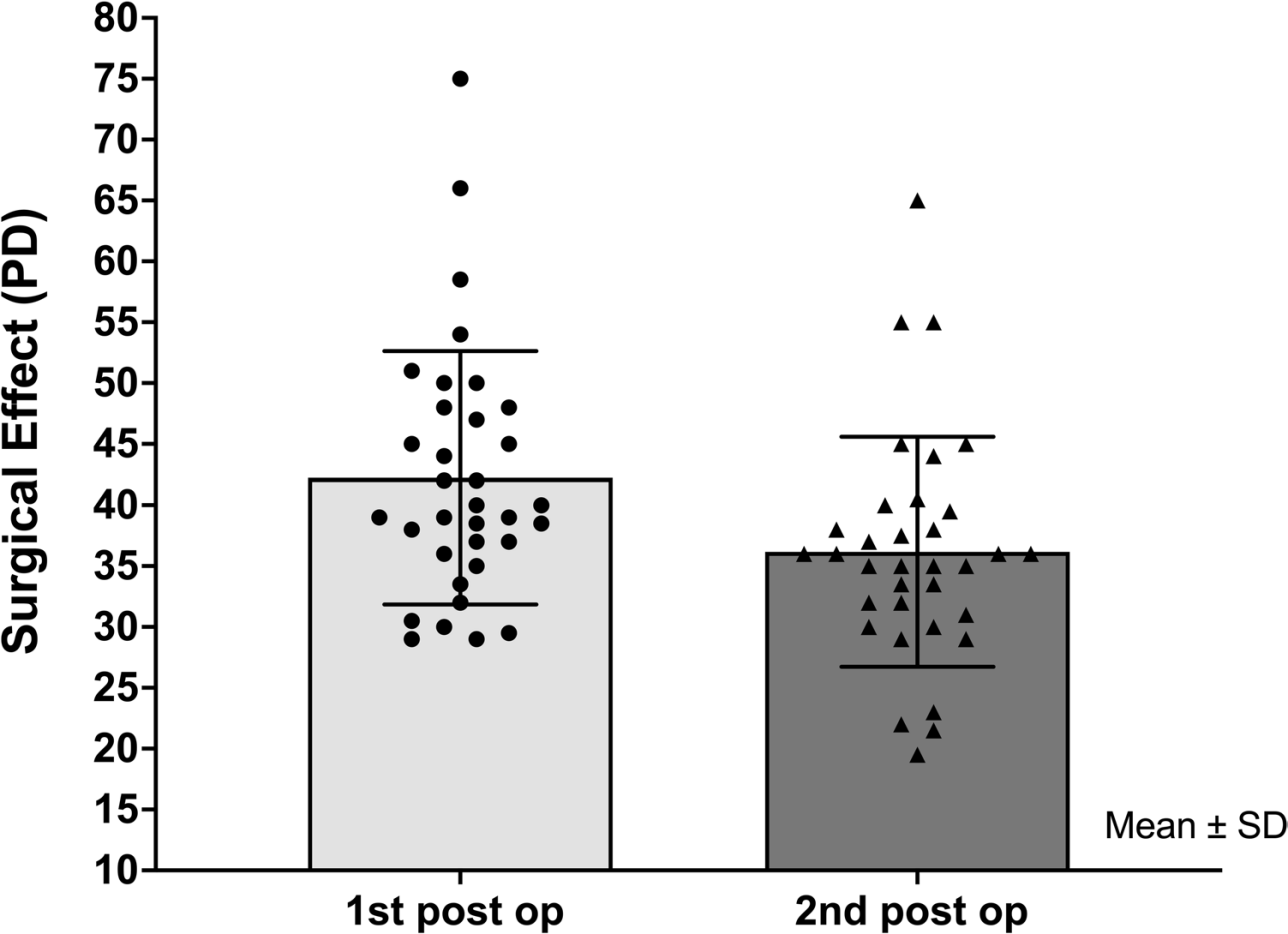
Surgical effect of bilateral rectus muscle recession 7 mm and unilateral rectus muscle plication 4 mm in exotropia. Surgical effect varied from 29Δ to 75Δ and from 22Δ to 55Δ at the first and second postoperative visita, respectively

Surgical effect varied from 29Δ to 75Δ, averaging 42 ± 10Δ at the first postoperative visit (Fig. 2). Exotropic shift occurred in most patients, so that the late surgical effect of the identical operation the second postoperative visit varied from 22Δ to 55Δ, but ultimately averaged 36 ± 8Δ. The mean ratio of effect to total surgical dose was 2 ± 1Δ/mm (range 2–4Δ/mm) at the first and 2 ± 1Δ /mm (range 1–3Δ/mm) at the second postoperative visit.

**Fig. 3:**
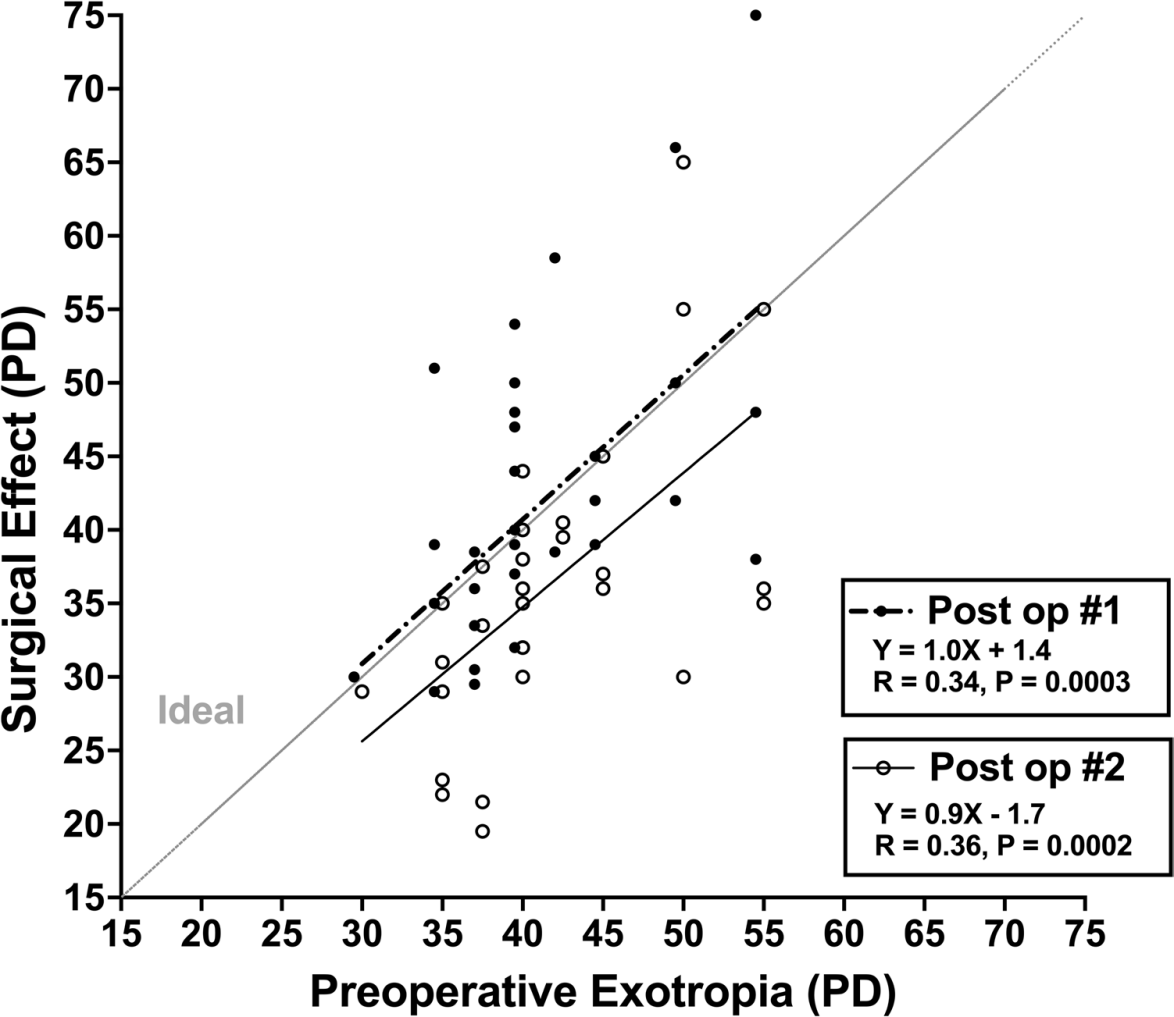
Correlation between preoperative exotropia and surgical effect. Preoperative deviation alone accounted for 34% and 36% of surgical effect at both first and second postoperative visit. Symbols of the first postoperative visit are nudged to the left to avoid overlap. The first post-operative visit occurred at an average of 6.9±4.1 (range 1–21) days after surgery, and the second at 60 ± 31 (range 22–183) days

Linear regression of surgical effect against pre-operative exotropia angle yielded a slope of 1.0 with 95% confidence interval (CI 0.49, 1.47) at first postoperative visit, and 0.9 with CI (0.47, 1.35) at the second postoperative visit (P < 0.001 for both), with a coefficients of determination (*R*^2^) of 0.34 and 0.36 at the first and second postoperative visit, respectively. There was no significant difference of the slopes between the first and second postoperative visits and neither of these slopes differ significantly from unity (P = 0.82, Fig. 3). This is consistent with the hypothesis that the average effect of the surgery is to eliminate whatever pre-operative exotropia exists. As indicated by the coefficients of determination, preoperative deviation accounted for 34-36% of variation in surgical effect at both first and second postoperative visits.

## Discussion

This study challenges conventional wisdom by repeating the identical strabismus operation in all included patients to demonstrate that the effect of strabismus surgery for moderate to large angle exotropia does not depend strongly upon surgical dose but on average corrects whatever exotropia exists. The surgical effect of 7mm bilateral lateral rectus muscle recession with 4mm unilateral medial rectus plication varied widely, from 29 to 75Δ, in treating exotropia ranging from 30 to 55Δ. The preoperative deviation alone accounted for 34–36% of variation in surgical effect, suggesting that surgical success for moderate to large angle exotropia is attributable more to the biological characteristics of the disease than to the amount of surgery performed. To our knowledge, this is the first clinical study that assessed the contribution of the preoperative range of exotropia to the surgical response in patients undergoing the same amount of surgery.

Archer compared the contributions of preoperative deviation and the amount of surgery as predictors of the response to esotropia surgery [24]. In Archer’s study, published strabismus surgery dose-response data were analyzed in a multiple linear regression model that included preoperative esotropia and the amount of surgery as variables. By analyzing the dose-response data presented by Tran et al. [23], Archer demonstrated that the partial correlation for the preoperative congenital esotropia angle exceeded that for the amount of bilateral medial rectus muscle recession. To evaluate the isolated correlation of preoperative deviation and the surgical outcome, Archer re-analyzed Scobee’s data on patients who all underwent same amount of surgery regardless of the amount of preoperative esotropia [25]. Linear regression revealed a coefficient of determination of 0.78, indicating that preoperative deviation alone accounted for 78% of the surgical effect of 6mm bilateral medial rectus muscle recession [24]. Based on these analyses, Archer concluded that biological adaptive response to strabismus surgery in congenital esotropia is more important than the mechanical effect of surgery.

Surgical dose-response curves or tables may provide general guidelines for strabismus surgery but cannot accurately predict surgical outcome. Identical surgery performed by the same surgeon in patients who have the same preoperative deviation often yields varying results. Experimental studies demonstrate several adaptive changes in extraocular muscles of strabismic animals and humans [26–29]. For example, strabismus surgical procedures such as recession and tenotomy are known to induce modulation of myosin expression and fiber remodeling in operated muscles [29].

In previous studies, three-muscle surgery has been mostly performed for exotropia exceeding 50Δ [17–22]. The average exotropia included in published studies ranged from 13 to 63Δ at distance, lateral rectus muscle recession ranged from 8 to 20 mm, and medial rectus muscle resection ranged from 5 to 9 mm [17–22]. Since there are no widely-accepted recommendations for dosing three-muscle surgery, dosage even for the same exotropia angle has varied among studies. Burian and Spivey recommend limiting lateral rectus muscle recession to 7 mm, and suggest adding unilateral medial rectus muscle resection when the exotropia is large [30]. The current study performed 7 mm bilateral lateral rectus recession with 4 mm unilateral medial rectus plication in all patients. The limitation of lateral rectus recession to 7 mm was reasoned by the location of the lateral rectus pulleys. Rectus muscle pulleys are located 8 mm posterior to the globe equator, so that recession of lateral rectus muscle beyond 7 mm induces acute lateral rectus slackness in the immediate post-operative period, altering the anterior force exerted by the lateral rectus pulley suspension [31]. Unilateral medial rectus muscle plication 4 mm was chosen to minimize the lateral incomitancy that can be induced by adding another horizontal muscle. We prefer plication to resection due to the former’s multiple advantages, including preserving the anterior ciliary vessels, possibility of early reversibility, absent risk of muscle loss, and less muscle trauma [32–34]; however, multiple studies suggest that plication and resection have quantitatively similar effects.

Depending on criteria for success and duration of follow up, previous studies report that success rates of three-muscle surgery for large angle exotropia varies widely from 42 to 85% [17–22]. Chen et al. report 42% success rate of three-muscle surgery in 34 patients who had 55Δ mean preoperative exotropia after a mean of 12 months; these authors state that greater exotropia angles are predisposed to undercorrection [22]. Nabie et al. report 85% success rate of bilateral lateral rectus muscle recession and unilateral medial rectus muscle resection in 48 patients having exotropic angles between 50 and 80Δ after 15 months mean follow up [21]. While our study had 76% success at 2 months mean follow-up, the shorter interval nevertheless suffices to answer the study question of whether the effect of three horizontal muscle surgery for moderate to large angle exotropia is better predicted by surgical dose or pre-operative angle. This study was performed using one specific surgical dosing regimen; it is possible that other fixed surgical dosing strategies might be similarly effective, or more or less effective, than the uniform strategy employed here.

This study has some limitations. Firstly, its retrospective design may introduce inherent biases including limitation only to patients who could be followed post-operatively. Although the data were retrospectively reviewed, a prospective decision on surgical dosing had previously been established. Secondly, the limited follow-up period precludes assessment of long term outcomes. Extended follow-up would provide long-term surgical outcomes, including success and recurrence rates, as well as the potential for late complications that did not emerge in the current study. However, this study did not aim to address long term changes beyond average of 10 weeks postoperatively. Surgeons were not masked when performing postoperative measurements.

In conclusion, the demonstrated correction of various angles of exotropia ranging from 30 to 55 Δ by the same amount of three-muscle surgery demonstrates that dose is not the main determinant of surgical effect in correcting moderate to large angle exotropia. This implies that clinicians need only measurements of precision sufficient to verify that pre-operative exotropia is in the range of 30–55Δ, as variations within this range lack important effect on surgical dosing and outcome. Bilateral lateral rectus recession 7 mm and unilateral medial rectus plication 4mm is effective in treating a wide range of moderate to large angle exotropia without causing significant lateral incomitance. Extended follow-up will be necessary to ascertain long term outcomes.
